# Limitations of Current Therapies and Barriers in Alzheimer’s Disease

**DOI:** 10.26502/aimr.0244

**Published:** 2026-05-27

**Authors:** Ayeshah Hussain, Iffat Alam, Devendra K. Agrawal

**Affiliations:** 1 Department of Translational Research, College of Osteopathic Medicine of the Pacific, Western University of Health Sciences, Pomona, California 91766 USA

**Keywords:** Acetylcholinesterase inhibitors, Alzheimer’s disease (AD), Amyloid-beta, Amyloid plaques, APOE4, Biomarkers, Blood-brain-barrier (BBB), Caregiver burden, Clinical trials, Cognitive decline, Diagnostic limitations, Disease-modifying therapies, Memantine, Microglial activation, Monoclonal antibodies, Neurodegeneration, Neurofibrillary tangles, Neuroinflammation, Neuronal loss, Symptomatic treatment, Synaptic dysfunction, Tau protein

## Abstract

Alzheimer’s disease (AD) remains a major global health crisis due to its complex pathophysiology and limited therapeutic effectiveness. Despite advances in understanding key mechanisms such as amyloid-beta accumulation, tau pathology and neuroinflammation, current therapies provide limited clinical benefit. Multiple factors contribute to limitations and highlight the difficulty of translating scientific advancements into meaningful improvement in patient outcomes. This article provides a comprehensive and critical review of therapeutic, biological, clinical, and systemic barriers to effective Alzheimer’s disease management as well as showcasing emerging strategies aimed to improve early detection, treatment approaches, and overall disease prevention.

## 1. Introduction

**Figure 1:**
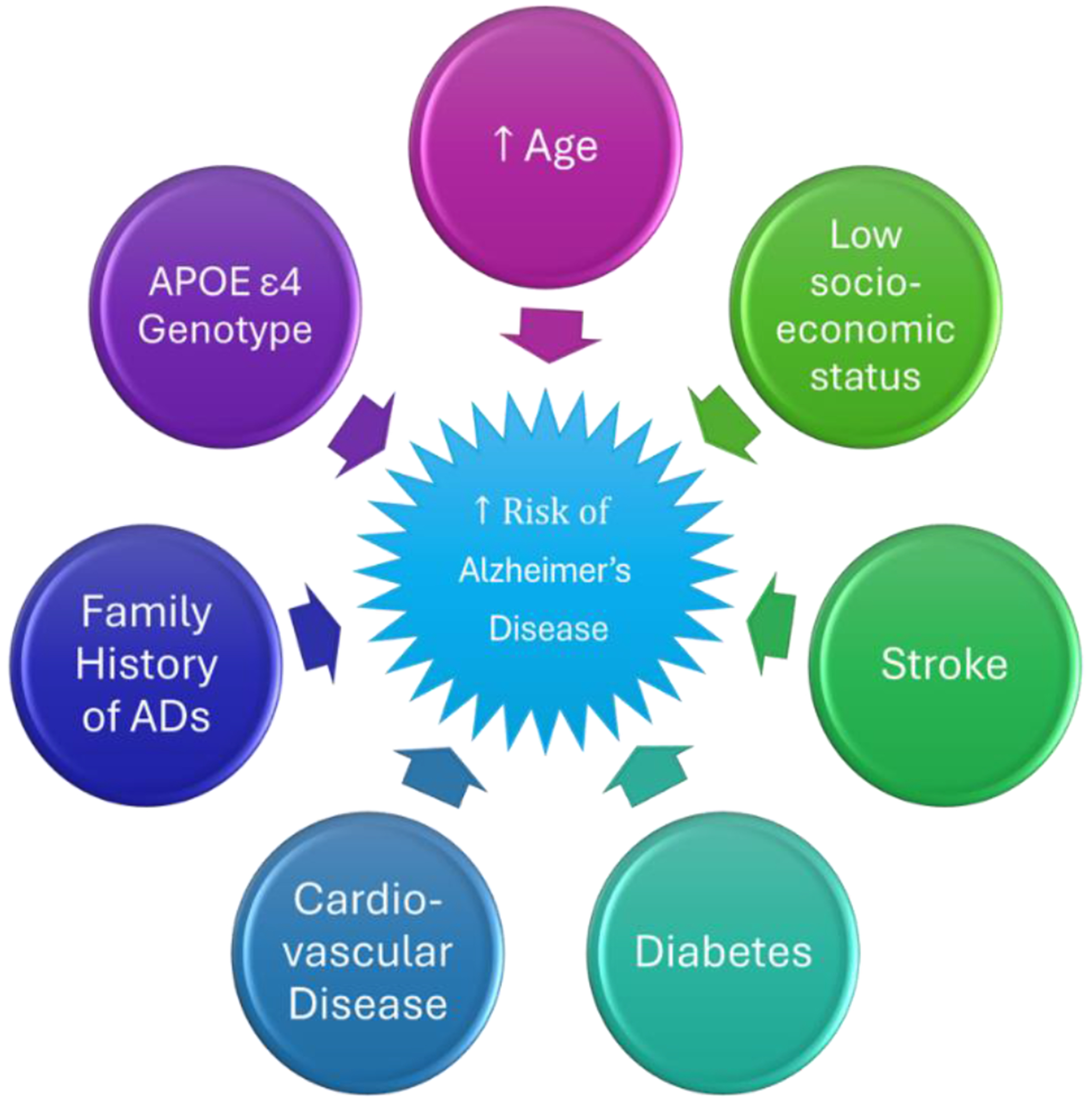
Risk factors in the pathogenesis of Alzheimer’s disease (AD).

Alzheimer’s Disease (AD) represents a growing global health crisis, with both prevalence and incidence increasing rapidly. Worldwide, the number of individuals affected is projected to rise from 30 million to over 106 million by 2050 [1]. In the United States alone, cases are estimated to rise from 5.8 to 13.8 million within the next 2 decades. Age is among the strongest and most significant risk factors for AD, with incidence increasing substantially in older populations (Figure 1). With an expected increase in life expectancy, the occurrence of AD is expected to grow correspondingly [2]. In addition to age, there are statistically significant findings that indicate differences in prevalence regarding sex. Women experience Alzheimer’s at rates approximating 1.17 times higher than those of men [3].

Genetic susceptibility also plays a critical role in AD, particularly through Apolipoprotein E ε4. (APOE4) allele. APOE4 is known as the strongest known genetic risk factor for Alzheimer’s disease [4–6]. Risk associated with APOE4 is dose-dependent with individuals carrying one allele having a 2-3-fold risk while homozygous carriers increase the risk of developing AD by 9-12-fold [4,5]. Because of its significance, APOE4 population distribution has been widely studied to assess variation in risk across various populations. Studies have shown that the APOE4 allele is most observed in East Asian and Caucasian populations compared to Black and Hispanic populations [6].

Atrial fibrillation not only affect the heart function but may also induce the progression of cognitive impairment and AD [7–11]. A TREM-2 variant has been associated with a significant increase in risk of developing AD [12]. There is also a crosstalk between protective and detrimental effects of IL-1 family cytokines in AD [13–15].

With such a high prevalence, Alzheimer’s disease also imposes a substantial economic burden. The global cost of dementia was estimated at nearly $1 trillion in 2015 and is projected to exceed $9 trillion by 2050 [16]. This estimate includes direct costs such as medical care and research as well as indirect costs that involve caregiving and loss of productivity. From a public health perspective, Alzheimer’s disease represents a major health crisis. Socioeconomic status has been shown to have a significant influence on cognitive outcomes. Research suggests that individuals with lower socioeconomic status have white matter hyperintensities, ultimately providing evidence that social and economic play a critical role in AD risk and progression [17].

## 2. Etiology and Pathophysiology

### 2a. Amyloid-beta pathology

Alzheimer’s disease is most notably characterized by the accumulation of amyloid-beta peptides in the brain that form extracellular neuritic plaques. These peptides are derived from the cleavage of amyloid precursor proteins (APP) and their accumulation is associated with a disruption is cellular homeostasis and progressive neuronal degeneration [18]. Aside from plaque formation, the presence of small, soluble, amyloid-beta oligomers plays a critical role in neurotoxicity. These oligomers disrupt synaptic function, impair neutral communication, and trigger downstream cascades that lead to tau aggregation, inflammation, and neuronal degeneration [19]. Under amyloid-beta induced neurodegeneration in vitro studies, astrocytes and microglia cells showed altered functions, including the production of neurotrophic factors which caused reactive cellular response. The cellular response led to an inflammatory environment that only accelerated neurodegeneration [20].

### 2b. Tau pathology

Tau pathology represents a central component of AD. It is defined as the abnormal aggregation of intracellular tau proteins. Under normal conditions, tau functions as a microtubule-associated protein that stabilizes neutral structures. However, under Alzheimer’s disease, hyperphosphorylation of tau proteins causes detachment from microtubules and leads to cytoskeletal instability and impaired neuronal transport [21,22]. Following detachment from microtubules, Tau begins to aggregate into helical filaments that form into intracellular neurofibrillary tangles that are strongly linked to neuronal degeneration [22]. These aggregates can also act as “seeds” that enable the spread of Tau aggregation between neurons that form distinct confirmations and are capable of self-replication and transcellular propagation, ultimately leading to disease progression and heterogeneity [23].

In addition to structural damage, Tau dysfunction causes various other neuronal disruptions. Tau-mediated silencing has been observed in mouse models, suggesting that tau pathology disrupts neuronal function prior to cell death. This results in impaired neuronal connectivity particularly in hippocampal and entorhinal regions which are critical for memory [24]. Furthermore, Tau dysfunction has been linked to broader neurodegenerative processes such as synaptic dysfunction and progressive neuronal loss [23,25]. Experimental models highlight association between tau dysfunction and hippocampal vulnerability, reinforcing its significant role in AD progression

### 2c. Neuroinflammation

Alzheimer’s disease pathogenesis is not only restricted to neuronal components but is also shown to have a strong association with the immunological response in the brain due to stroke, traumatic brain injury, and other neurodegenerative diseases [26–32]. Neuroinflammation is primarily mediated by the activation of microglia and astrocyte cells [32–35]. In response to amyloid-beta accumulation, they release cytokines that contribute to neuronal damage [26]. While the inflammatory response is initially protective, prolonged and dysregulation periods can sustain extreme neurodegeneration [36]. At a cellular level, amyloid-beta accumulation is shown to directly cause astrocyte mitochondrial dysfunction. A lack of energy production ultimately leads to impaired brain homeostasis and exacerbation of AD progression [37].

Apart from glial activation, NLRP3 inflammasome pathway is also linked to Alzheimer’s pathology. NLRP3 is a major regulator of inflammatory mediators that drive neuronal injury and cause cognitive decline [38–42]. Experimental models indicate that activation of this inflammatory pathway is widely associated with Alzheimer’s disease progression by furthering the role of neuroinflammation as a central mechanism in AD pathogenesis.

## 3. Current Therapies

### 3a. Symptomatic therapies

**Figure 2:**
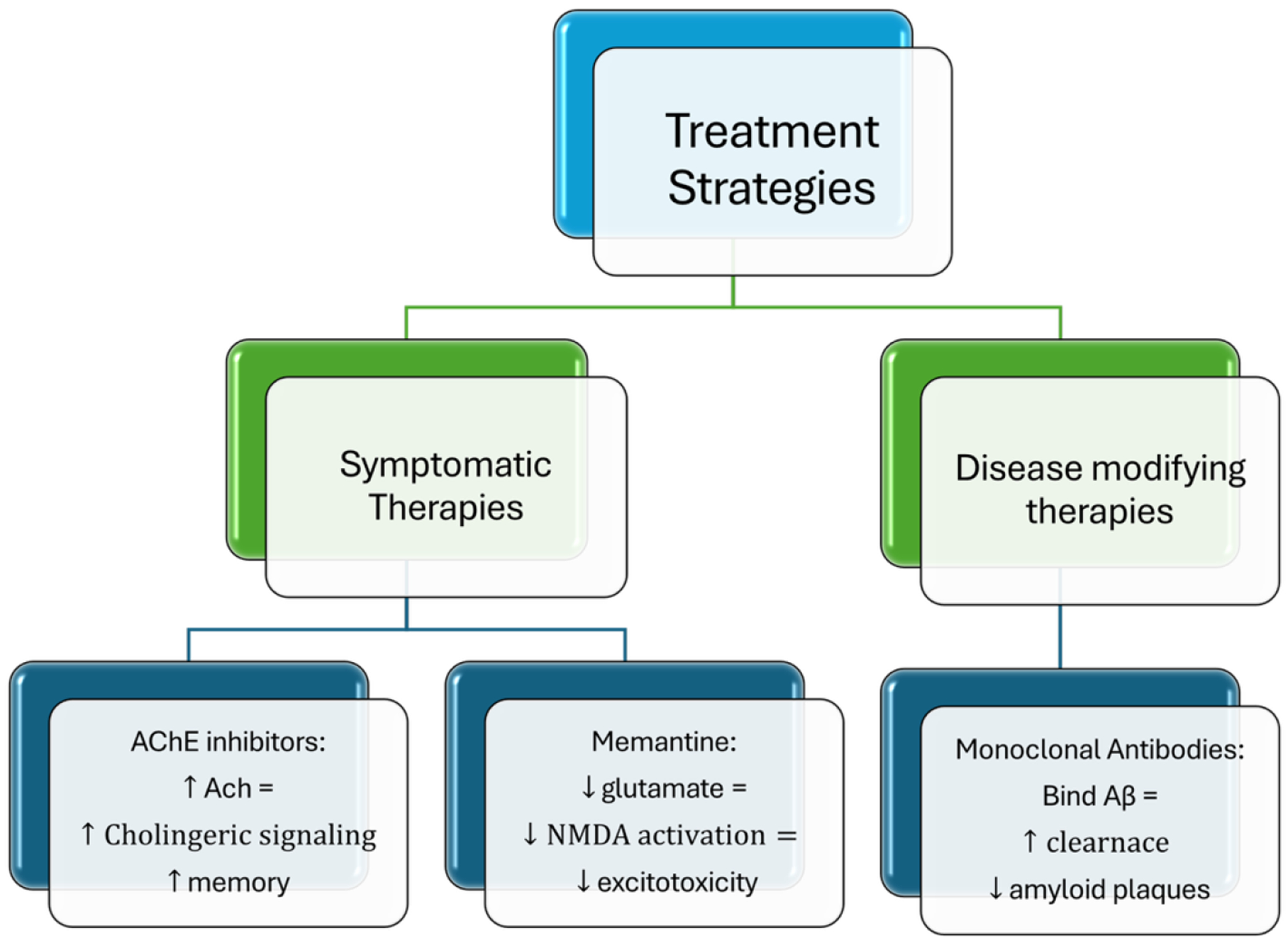
Treatment strategies in Alzheimer’s disease. These include symptomatic therapies and disease modifying therapies. Aβ, amyloid-beta; Ach, acetylcholine; NMDA, N-Methyl-D-Aspartate.

Pharmacological therapies for Alzheimer’s diseases are primarily symptomatic and aim to improve cognitive function but are limited when it comes to altering the underlying pathology of AD. Currently, the most used therapies are acetylcholinesterase inhibitors and NMDA (N-Methyl-D-Aspartate) receptor antagonist, memantine [43] (Figure 2).

In individuals experiencing AD, acetylcholine is significantly reduced, causing significant memory and cognitive dysfunction. Acetylcholinesterase inhibitors such as donepezil, galantamine, and rivastigmine work by inhibiting the enzyme that functions to break acetylcholine [44]. Increasing availability of acetylcholine at synapses leads to improvements of cognition and memory function, although the magnitude of improvement is limited in most patients [45].

In contrast, Memantine targets glutamatergic signaling by acting as a non-competitive NMDA receptor antagonist [46]. A reduction in excessive receptor activation correspondingly reduces excitotoxic neuronal damage. Memantine works by modulating abnormal glutamate activation by preserving normal synaptic transmission, proving to be especially effective in moderate to severe stages of AD [46].

Symptomatic therapies are typically used in combination depending on disease severity and progression [43] and work to provide modest improvements in cognitive and functional outcomes, though their effects are supportive rather than disease modifying [44,45].

### 3b. Disease modifying treatments

Recent therapeutic developments in AD have shifted towards disease modifying approaches that target underlying pathology, with many focusing on amyloid-beta accumulation in the brain. Monoclonal antibodies such as aducanumab, lecanemab, and donanemab work by selectively binding to aggregated forms of amyloid-beta (Figure 2). Ultimately, these therapies facilitate immune-mediated clearance and reduce overall plaque burden [47–52]. Positron emission tomography provides amyloid imaging that supports evidence that these therapies work to reduce amyloid plaque deposits through microglial activation [53]. Multimodal artificial intelligence approaches have been used the early diagnosis of neurodegenerative diseases [54]. These therapeutic approaches directly target underlying pathology of Alzheimer’s disease.

Phytochemicals, vitamin D, and other nutritional elements interact with apolipoprotein E ε4 and inflammatory cytokines to attenuate inflammatory response [55–58]. Indeed, higher concentrations of vitamin D and antioxidants are associated with better cognitive outcomes and thus helpful in Alzheimer’s disease [59]. Semaglutide can induce anti-inflammatory effects and thus has potential as a therapeutic strategy in Alzheimer’s disease [60,61].

Clinical trials have shown that these therapies produce biological and clinical changes. Lecanemab treated individuals have been found to have slower cognitive decline, especially when treated within early stages [62,63]. Donanemab has also demonstrated significant reductions in amyloid deposition and may represent improvements in cognitive and functional outcomes in clinical trials [49].

### 3c. Clinical considerations in Treatment Selection

In clinical practice, selecting patients for pharmacological and non-pharmacological interventions is primarily determined by the severity and presentation of symptoms. Pharmacological therapies are typically reserved for individuals presenting with more severe stages of cognitive impairment or significant behavioral symptoms of dementia [63,64]. In contrast, non-pharmacological strategies including cognitive stimulation, environmental modifications, and behavioral interventions are primary approaches for managing mild cognitive and behavioral symptoms. The reason for this is to avoid adverse effects of medications while still providing improved patient outcomes [65]. More specifically, non-pharmacological outcomes have been shown to reduce agitation, mood swings, and caregiver burden which makes them especially valuable in early-stage diseases [66]. These approaches are often implemented with pharmacological treatments or may be used alongside medication as symptoms progress, allowing for a more individualized treatment strategy [65].

## 4. Limitations of Current Therapies

### 4a. Limitations of symptomatic treatment

Although current symptomatic therapies provide measurable clinical benefits, their overall impact on AD progression remains limited. Acetylcholinesterase inhibitors and memantine have been shown to produce only modest improvements in cognitive function with many patients experiencing minimal or variable responses to these therapies [44]. While some individuals demonstrate slight improvement or short-term stabilization, these effects do not translate into substantial long-term functional gains [66].

A most major limitation of symptomatic treatments is the inability to alter underlying disease processes. These medications primarily target neurotransmitter systems rather than pathological mechanisms driving ADs and therefore, are ineffective when it comes to slowing neurodegeneration [43]. As a result, disease progression continues despite ongoing treatment. Additionally, symptomatic therapy benefits tend to diminish as Alzheimer’s disease progresses. Some research studies suggest that these treatment effects are more apparent in earlier stages and efficiency decreases in individuals with more advanced stages of ADs [45]. This stage-dependent response further limits long-term clinical utility as patients experience worsening symptoms despite continued therapy [66].

Adverse effects of symptomatic treatments also contribute to therapy limitations. Cholinesterase inhibitors are associated with gastrointestinal disturbances, bradycardia, syncope and several other side effects. These effects reduce tolerability and lead to discontinuation in some patients [68–73]. A combination of these factors alongside limited efficacy, represent significant barriers to sustained therapeutic benefit in AD management.

### 4b. Limitation of disease modifying therapy.

Amyloid-targeting therapies represent a significant advancement in AD treatment, but several limitations remain. Clinical trials have shown that monoclonal antibodies such as lecanemab and donanemab produce statistically significant but relatively small improvements in cognition, raising questions about the magnitude of their clinical benefits [62]. While reductions in amyloid-beta plaques are consistently observed, these changes translate to limited meaningful improvement in memory and cognitive functions.

A major safety concern with these therapies is the occurrence of amyloid-related imaging abnormalities (ARIA) which include complications such as cerebral edema and microhemorrhages. These adverse effects have been observed in a substantial proportion of treated patients and are thought to result from the removal of vascular amyloid deposits [63]. The high incidence of these complications therefore requires patients to undergo frequent magnetic resonance imaging monitoring to detect potential complications.

Aside from safety concerns, the long-term outcomes of amyloid-targeting therapies remain uncertain. While short-term studies provide evidence of reduced amyloid-beta levels and modest improvements in cognitive function, sustained benefits over extended periods of time are limited [50]. In various research studies, the relationship between amyloid plaque reduction and clinical improvement sparks debate as to whether lowering amyloid labels directly translates into meaningful therapeutic outcomes [alexander].

## 5. Biological Barriers to Effective Treatment

The underlying pathology of Alzheimer’s disease presents significant challenges in creating effective therapies. One major barrier is the multifactorial nature of the disease itself. Many therapies focus on a single target even with evidence suggesting that AD revolves around a combination of amyloid-beta accumulation, tau pathology, neuroinflammation, and metabolic dysfunction [68,69]. Therefore, formulating treatment plans requires targeting and modifying a combination of these factors.

Drug delivery constraints further complicate therapeutic development. The blood brain barrier (BBB) essentially protects the central nervous system by restricting the entry of many therapeutic agents into the brain. With the few therapies that work to cross the BBB, researchers are faced with the challenge of dealing with insufficient concentrations at target sites [70]. In addition, AD presents with variable heterogeneity across affected individuals. Variations in genetic risk factors and underlying mechanisms lead to the need of complex therapeutic differences and limits the effectiveness of uniform treatment plans [73].

## 6. Clinical and Diagnostic Challenges

### 6a. Diagnostic limitations

Alzheimer’s disease is characterized by a prolonged preclinical phase which consists of pathological changes despite a lack of observable symptoms. Individuals may remain cognitively normal while accumulating AD pathology, with symptoms only emerging in later stages [74]. As a result, clinical diagnosis typically occurs after significant neurological damage has already occurred, limiting the effectiveness of therapeutic interventions.

Moreover, current diagnostic methods present practical limitations. Cerebrospinal fluid biomarkers including amyloid-beta and tau proteins both provide valuable insight into underlying pathology [75], but their use requires lumbar puncture, an invasive procedure that limits widespread screening and early detection [76].

### 6b. Clinical trial challenges

Clinical trials for AD therapies face several methodological challenges that further complexify its evaluation of therapeutic efficacy. One limitation is late patient enrollment. Many trials include patients that have already reached symptomatic stages which may signify a point of irreversible neurodegeneration [77]. Intervention at this late stage reduces the likelihood of detecting meaningful treatment effects.

The use of cognitive endpoints presents another challenge in clinical trial outcome assessment. Commonly used measures lack sensitivity to detect small changes in cognitive function and may not accurately reflect meaningful clinical improvements [78]. This leads to difficulty in interpreting whether observed changes are true therapeutic benefits. Additionally, short-term durations of many clinical trials limit the ability to assess long-term treatment effects in AD progression. Alzheimer’s disease develops gradually causing short trials to fail to show full impacts of intervention on disease trajectory [52].

Finally, high placebo response rates present a major obstacle in AD clinical trials. Variability in cognitive performance and disease progression among placebo groups make it difficult to accurately assess true outcomes. Studies have shown that patients receiving placebo may still demonstrate measurable changes in cognitive scores over time, highlighting the fluctuating nature of AD and the limitations of available assessment methods [45,79]. This variability may obscure true drug efficacy and reduce the ability of clinical trials to detect differences in treatment and control groups.

## 7. Socioeconomic Barriers

Alzheimer’s disease presents significant systemic and economic barriers that limit effective diagnosis and treatments. The financial burden associated with ADs, is substantial and continues to rise globally. In 2015, the annual cost per patient was estimated to be $19,144 with total cost reaching $957.56 billion worldwide. These costs are estimated to rise to $2.54 trillion by 2030 and $9.12 trillion by 2050 [16]. Most of these costs are also not limited to medical expenses. Studies have found that indirect costs make up more than 50% of total cost [16]. This includes monetary loss from inability to work, reduced caregiving income, and treatment of caregiving burden and injuries [16]. These findings indicate that AD burden extends beyond clinical treatment and has major effects on long-term stability, ultimately delaying diagnosis especially for patients with limited financial resources.

Systemic inequalities further contribute to challenges in getting patients treatment. Research has shown that Alzheimer’s disease clinical trials and treatment pathways often underrepresent minority populations with up to 94.7% of clinical trial patients being of Caucasian descent [80]. Strict eligibility criteria for clinical trials such as caregiver involvement can limit access to diagnosis and treatment for lower socioeconomic status patients [80]. These disparities reduce generalizability of clinical trial findings and lead to unequal access to emerging therapies.

Caregiving burden is an additional barrier that impacts both diagnosis and treatment. With chronic illnesses such as AD, patients require long-term, intensive care. Caregivers experience significant physical and emotional strain as well as financial burdens that can all interfere with their ability to effectively manage care [81]. Caregivers have the responsibility of coordinating appointments, administering and ensuring adherence to medication/care, and monitoring symptoms. Caregiver burden can lead to delayed therapeutic intervention and reduce quality of care. Together these factors create barriers to effective disease management, even when diagnostic and therapeutic practices are available.

## 8. Emerging Solutions

Recent advancements in Alzheimer’s have shifted toward therapies targeting multiple pathological pathways rather than focusing on a single mechanism. Several research efforts have started to focus on a more comprehensive understanding of disease progression [77]. These combination therapies overcome limitations of single-target approaches [69] and represent a shift towards more biologically integrated treatment models.

Additionally, Gene-based therapies have been a primary focus within the past decade. Genetic risk factors such as the APOE variant are being regularly used to assess differences in disease susceptibility and response to treatments [72,73]. These therapies focus on targeting everyone’s unique genetic profile to improve treatment efficacy and reduce onset/progression [74–76].

Early detection of AD has also been a major focus of research to prompt early intervention and decrease disease progression. Biomarker development advances using imaging and fluid markers allow for detection of pathological changes in asymptomatic individuals [78]. Efforts have shifted towards the preclinical stage of AD at which intervention remains to be the most effective [52].

Lifestyle modifications have also been found to play a role in reducing the risk of Alzheimer’s disease According to various studies, 30-40% of AD diagnosis may be attributable to modifiable risk factors such as cardiovascular disease, lack of physical activity, and low educational attainment [1,66]. Lifestyle modifications that focus on improving overall health and preventing AD risk factors delay disease onset.

Although these therapeutic trials are still within early stages, they present a promising direction for better targeted therapy and AD management.

Improving access and economic barriers for AD patients is also becoming a promising effort. Given the rapid rise in global cost of AD [16], substantial effort and funding have been directed towards clinical research and trials within the last decade. Researchers are working to expand access to diagnostic tools, increase diversity in clinical trials, and develop therapies that prove efficacy in broader populations of affected individuals [82]. Addressing these challenges will be essential for translating into meaningful improvements in Alzheimer’s disease therapy.

## Key points

- Alzheimer’s disease incidence rate is increasing rapidly driven by an aging population and rising life expectancy.
- Differences in Alzheimer’s disease prevalence across racial and ethnic groups suggest variation in genetic susceptibility and possible social determinants of health contribution to disease risk.
- Amyloid-beta accumulation, tau pathology, neuroinflammation collectively contribute to Alzheimer’s disease progression and cognitive decline.
- Current symptomatic therapies provide modest improvements in cognition but do not address underlying pathological mechanisms.
- Disease-modifying therapies reduce amyloid–beta plaque but provide limited clinical benefits in terms of improving cognitive outcomes.
- The long preclinical phase of Alzheimer’s diseases plays a major role in late diagnosis and intervention.
- Clinical trial challenges involve high placebo rates and limitations in accurately measuring meaningful findings of cognitive improvements.
- Diagnostic limitations include invasive testing methods such as lumbar taps and suggest variability in biomarker accuracy.
- Economic burden, underrepresentation of minority populations in clinical trials, and caregiver burden significantly limit accessibility to the treatment of Alzheimer’s disease.
- Emerging strategies of treating Alzheimer’s disease involve focus on early detection, lifestyle interventions, reduced cost, and combination therapy.
